# Measurement of Maize Leaf Phenotypic Parameters Based on 3D Point Cloud

**DOI:** 10.3390/s25092854

**Published:** 2025-04-30

**Authors:** Yuchen Su, Ran Li, Miao Wang, Chen Li, Mingxiong Ou, Sumei Liu, Wenhui Hou, Yuwei Wang, Lu Liu

**Affiliations:** 1 School of Engineering, Anhui Agricultural University, Hefei 230036, China; yuchensu@stu.ahau.edu.cn (Y.S.); ranmaxli@stu.ahau.edu.cn (R.L.); shmilywm@stu.ahau.edu.cn (M.W.); lichen111@stu.ahau.edu.cn (C.L.); liusm1117@ahau.edu.cn (S.L.); hwh303@ahau.edu.cn (W.H.); wyw@ahau.edu.cn (Y.W.); 2 High-Tech Key Laboratory of Agricultural Equipment and Intelligence of Jiangsu Province, Jiangsu University, Zhenjiang 212013, China; myomx@ujs.edu.cn; 3 Wandong Comprehensive Experimental Station, Anhui Agricultural University, Chuzhou 239400, China

**Keywords:** maize phenotype, 3D point cloud, clustering segmentation, stem and leaf segmentation, digital modeling

## Abstract

Plant height (PH), leaf width (LW), and leaf angle (LA) are critical phenotypic parameters in maize that reliably indicate plant growth status, lodging resistance, and yield potential. While various lidar-based methods have been developed for acquiring these parameters, existing approaches face limitations, including low automation, prolonged measurement duration, and weak environmental interference resistance. This study proposes a novel estimation method for maize PH, LW, and LA based on point cloud projection. The methodology comprises four key stages. First, 3D point cloud data of maize plants are acquired during middle–late growth stages using lidar sensors. Second, a Gaussian mixture model (GMM) is employed for point cloud registration to enhance plant morphological features, resulting in spliced maize point clouds. Third, filtering techniques remove background noise and weeds, followed by a combined point cloud projection and Euclidean clustering approach for stem–leaf segmentation. Finally, PH is determined by calculating vertical distance from plant apex to base, LW is measured through linear fitting of leaf midveins with perpendicular line intersections on projected contours, and LA is derived from plant skeleton diagrams constructed via linear fitting to identify stem apex, stem–leaf junctions, and midrib points. Field validation demonstrated that the method achieves 99%, 86%, and 97% accuracy for PH, LW, and LA estimation, respectively, enabling rapid automated measurement during critical growth phases and providing an efficient solution for maize cultivation automation.

## 1. Introduction

Maize is the most widely planted food crop in the world and plays an important role in ensuring national food security [1]. In recent years, researchers have integrated intelligent sensing and digital technologies with agricultural development, injecting new momentum into agricultural modernization [2,3,4]. Automation of the planting process is critical for the yield of maize, which requires the measurement of morphological parameters relating to plant growth status [5]. Among different parameters, plant height (PH), leaf width (LW), and leaf angle (LA) of maize have been reported to have the capability to indicate the growth status and yield of maize [6]. Therefore, automatic acquisition of these parameters is crucial for the research of precision agriculture and cultivation of maize [7].

Traditional manual measurement of maize stem and leaf parameters is labor-intensive, inefficient, and error-prone [8]. With the development of sensing and computing technology, the morphological parameters of maize plants, such as PH, LW and LA, can be quickly obtained with customized algorithms using the data collected by the sensor [9]. At present, in a complex environment, the three-dimensional information of crops is mainly obtained by using three different sensing technologies, that is, stereo camera, depth camera, and lidar [10].

A stereo camera, which simulates the observation process of human eyes, can capture plant information from multiple perspectives [11]. Ma et al. used a camera to obtain RGB images of maize, based on which the geometric relationship between shooting angles and measured image pixels were investigated to obtain the HP, LW, and LA values [12]. Dimitris Zermas et al. first used a camera to shoot a large number of high-resolution RGB images, reconstructed a three-dimensional point cloud model of maize through multi-image analysis, and lastly measured the PH and LA values of maize [13]. Raju et al. used a Raspberry Pi camera to take photos of maize during different growth periods and developed an image processing framework based on MATLAB 2022b to measure the LA value of maize. Despite the potential of the stereo camera, its performance will be greatly affected when the light is strong and dark. Thus, the depth camera is utilized to solve this kind of problem [14]. Chaivivatrakul et al. collected the point cloud data of maize with a TOF camera and measured the LA value through plane fitting [15]. What is more, Lu et al. made use of the collected point cloud to extract the maize stem by Z0Y plane projection and measured leaf length (LL) and LA values of maize on the plane [16]. However, the depth camera also maintains some disadvantages that affect its outdoor application, such as narrow measuring range, small field of vision, easy interference by sunlight, and inability to measure transmission materials. Therefore, the depth camera is mainly used in indoor environments.

Laser radar uses a laser as the transmission signal for environmental sensing, which has the advantages of high resolution, high sampling rate, good concealment, strong anti-interference ability, good low-altitude detection performance, and portability [17,18]. Due to the superior advantages over other sensing technologies, lidar has become the most popular choice for accurate measurement of PH, LW, and LA. After obtaining the point cloud of a target plant, Liang et al. first corrected the stem direction of a single maize plant. Then, the random sampling consistency algorithm was adopted to segment the maize stem. Subsequently, the conditional Euclidean clustering method was used to extract the stem point cloud to segment a single leaf. Finally, plant phenotypic information such as plant height, stem diameter, and leaf area of maize was obtained [19]. Teng et al. extracted the skeleton of maize seedlings, roughly segmented the skeleton, and finely segmented the stem and leaf [20]. Lai et al. used a Euclidean clustering algorithm to segment plant population, regional growth algorithm, edge extraction algorithm, hypervoxel clustering algorithm, and the concave–convex method to segment different plant leaf organs, and regional growth clustering method was used to segment maize leaves [21]. Zhu et al. segmented the point cloud of maize plants by hypervoxel clustering and local features, and then counted the point cloud density of maize tassels [22]. To solve the problem that terrestrial laser scanning (TLS) is rarely used in field crop phenotype research, a method of extracting field maize phenotype by TLS was developed by Su et al. On this basis, the leaf skeleton of maize plant was first extracted, and the leaf length, leaf area, plant height, and leaf inclination of maize were then measured [23]. Qiu et al. put forward a method of collecting phenotypic data of large plant groups by installing a VLP-16 lidar on a mobile robot and measuring the plant spacing and plant height of maize [24]. Aiming at the problem that it is difficult to accurately estimate the phenotypic parameters of the whole growth cycle of maize plants from 3D point clouds, Wu et al. developed a method of point cloud clustering and noise reduction to extract the skeleton and measure the leaf length, leaf inclination, leaf apex length, leaf azimuth, leaf growth height, and plant height of maize [25]. Ao et al. used ground lidar combined with convolutional neural networks (CNNs) and morphological characteristics to cut the stems and leaves of a single maize plant in a field environment to measure the plant height, crown width, and leaf area of maize [26]. Jin et al. used the median normalized-vector growth (MNVG) algorithm to segment maize stems and leaves, and measured leaf inclination, leaf length, leaf width, stem height, and stem diameter [27]. Miao et al. collected maize point cloud data by ground laser scanning, segmented the stems and leaves of maize by point cloud registration, ground segmentation, filtering noise reduction, and Euclidean clustering, and measured the stem length and short-axis thickness by ellipse fitting [28]. Liu et al. used ground VLP-16 lidar to collect the point cloud data of maize in a basin, segmented the stem and leaf tissues of maize based on the regional growth algorithm and Euclidean distance, and obtained phenotypic parameters of maize such as leaf length, leaf width, leaf area, and leaf angle through spatial coordinate projection and shortest path distance [29]. Li et al. employed a rail-based field phenotyping platform integrating lidar and RGB cameras to collect multi-source data, achieving point cloud fusion and plant segmentation through direct linear transformation algorithm, time-series image-guided registration, cloth simulation filter, and region growth algorithms. They demonstrated that multi-source data fusion significantly improved time-series phenotype extraction accuracy [30]. Chen et al. proposed a point fractal network-based completion method combined with single-view RGB-D image deep learning reconstruction and triangular mesh leaf area parameter extraction. Their results showed significant improvement in occluded flowering Chinese cabbage point cloud completion and phenotype parameter accuracy, offering a novel approach for non-destructive plant phenotype identification [31]. Noshita et al. developed a 2D–3D fusion method integrating deep learning-based instance segmentation, structure from motion, and B-spline curve fitting with leaf correspondence identification and curve fragment assembly. Their results demonstrated effective 3D leaf edge reconstruction, while highlighting challenges in small leaves or high-camera-noise scenarios, offering a non-destructive approach for quantitative plant morphological analysis [32].

The method of collecting maize point cloud by fixing VLP-16 lidar in the field has the disadvantages of long collection time, low data collection efficiency, and great environmental impact, so it is difficult to collect three-dimensional point cloud information of crop plants in the field. VLP-16 lidar has longer measured distance and higher data acquisition efficiency and can quickly acquire three-dimensional [33] point clouds of field crops. VLP-16 lidar can be used to measure the PH, LW, and LA of crops, but it has the following problems. The PH value is mostly defined as the distance from the highest point of crops to the ground, but there will be errors in the measurement of PH because the maize in the field does not grow perpendicularly to the ground. Stem–leaf segmentation is usually realized by cylindrical segmentation, which leads to inaccurate stem–leaf segmentation and then affects measurement accuracy. In addition, a point cloud extracted at a fixed height may be located at the junction of leaves and stems, resulting in inaccurate measurement of position. It is necessary to study a new method of PH measurement in terms of how to determine the measurement position of LW and LA of leaves through stem–leaf segmentation.

To solve the above problems, VLP-16 lidar was used to collect point cloud data of different growth stages of maize, and the PH, LW, and LA of maize were measured based on the point cloud data. A PH measurement method for calculating the vertical distance from the highest point to the root of maize plants was proposed. A method combining spatial projection with normal distribution was proposed to segment maize stems and leaves. After identifying leaf point clouds, LW and LA were obtained by spatial projection [34], edge detection, and linear fitting.

## 2. Materials and Methods

### 2.1. Architecture of the System

**Figure 1:**
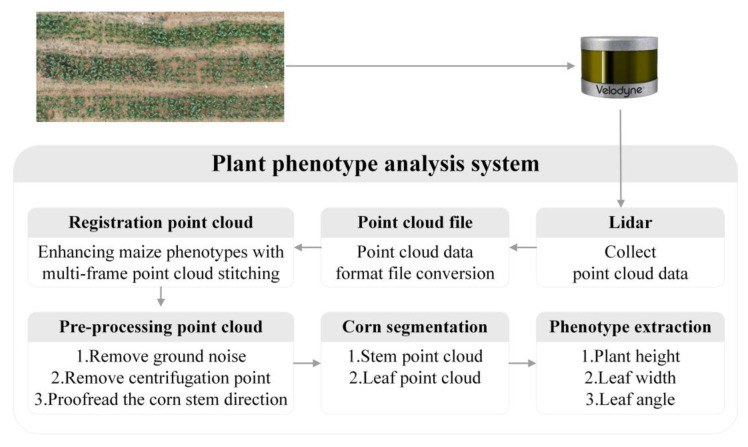
Overview of the system, including screening system, modeling system, and main steps in segmenting system.

The architecture of our proposed plant phenotype system is shown in Figure 1. Taking maize plants as the research object, the PH, LW, and LA of maize were measured. Firstly, VLP-16 lidar was used to collect the three-dimensional point cloud data of maize plants during the middle and late growth stages. The specifications of VLP-16 lidar (Velodyne, San Jose, CA, USA) are listed in Table 1. Then, to enhance the phenotypic information of maize plants, a point cloud registration method is introduced to fuse the information from multiple frames. After that, the ground points and other outliers are filtered to obtain the point clouds of the plant area of interest. Finally, point cloud data processing methods are proposed for the automatic measurement of PH, LW, and LA of maize. To evaluate the performance of the proposed system, the automatic measuring results are compared with manually measured PH, LW, and LA values.

### 2.2. Data Collection

**Figure 2:**
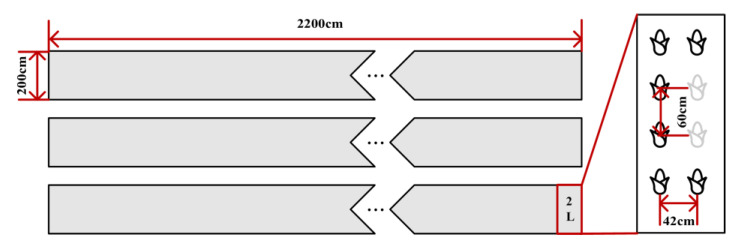
Maize planting map.

The experimental site of data collection was an experimental field in Baohe District, Hefei City, Anhui Province (coordinates: 117.370585, 31.796655), which is 2200 cm long and 700 cm wide. The variety of maize is Jinyunuo 856. There are three plots of maize with a spacing of 50 cm, and each plot has four rows and 50 columns. Maize from middle and late stages with plant spacing of 60 cm was taken as the research object, as shown in Figure 2. The experiment period was from 30 March to 30 June 2022. The data were collected from 1 May to 30 June 2022.

**Figure 3:**
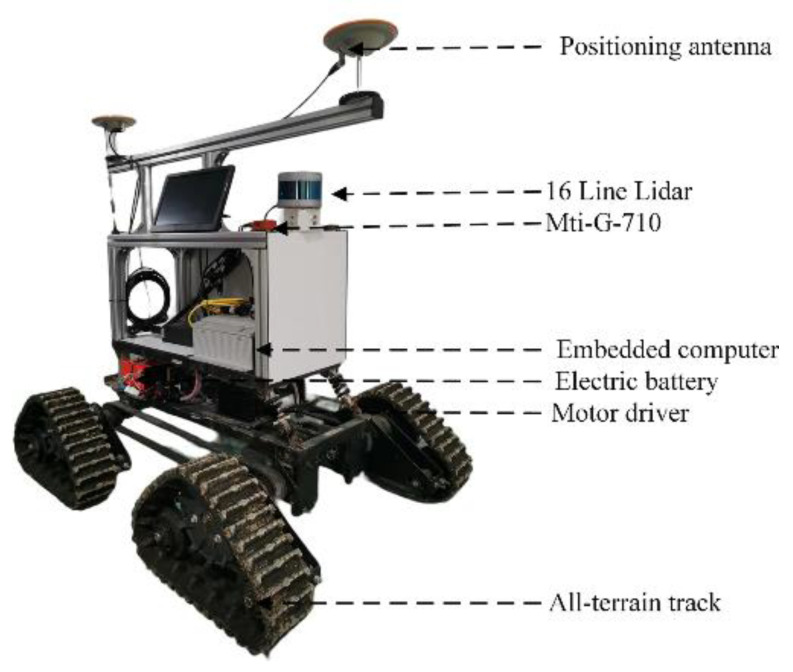
Maize sensing vehicle platform.

A VLP-16 lidar (Velodyne) was used to collect the three-dimensional point cloud data of maize plants in the middle and late stages. In addition, the VLP-16 lidar was installed on top of a self-made moving platform to collect the three-dimensional point cloud data of maize plants. The general configuration of the sensing platform is shown in Figure 3. At the top of the platform is a GNSS integrated navigation and inertial measurement unit for vehicle positioning and information tracking, and the middle of the platform is equipped with an embedded computer to store the collected field point cloud information. Moreover, to meet the driving requirements of different roads, the bottom of the platform is equipped with a triangular crawler wheel.

### 2.3. Three-Dimensional Point Cloud Data Processing

**Figure 4:**
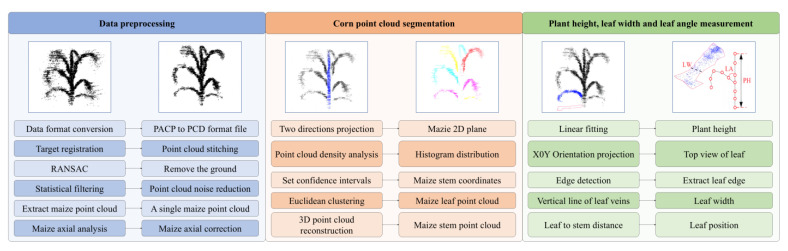
Overall point cloud data processing block diagram.

The Velodyne Velo View and Cloud Compare software packages (CloudCompare_v2.11.0_bin_x64 and VeloView-3.1.1-26022015-Windows-64bit) were used as three-dimensional point cloud data processing tools to extract point cloud data. C++ language was used to realize the sampling reduction of point clouds, and a MATLAB program was developed to measure the PH, LW, and LA values of maize. A block diagram of the overall data processing procedure is shown in Figure 4, which includes three parts, that is, data preprocessing, maize point cloud segmentation, and phenotypic parameter measurement. The challenge of maize point cloud processing is how to quickly realize stem–leaf segmentation. The usual method is to convert point clouds into an image, then identify the image boundary and perform ellipse fitting, and finally realize the stem–leaf segmentation of point cloud. To solve this problem, we proposed a fast segmentation method of maize stems and leaves by combining spatial bidirectional projection with normal distribution.

#### 2.3.1. Data Preprocessing

The data preprocessing process includes point cloud data format conversion, point cloud data splicing, ground point cloud elimination, noise reduction, maize single-plant segmentation, and maize stem axial correction.

Step 1: Velodyne Velo View software was used to read the collected data frame by frame. In addition, to improve the efficiency of data processing and adapt to the application of Point Cloud Library (PCL), a program was designed to convert the three-dimensional point cloud data from *.PACP format to *.PCD format.

Step 2: The point density of the data in a single frame is sparse, from which it is impossible to obtain complete phenotypic information of maize. To obtain the complete information of the measured maize, 100 frames of point clouds were selected, and the Gaussian mixed model algorithm was used to register the point clouds.

Step 3: Since the collected data contain complex environmental information such as ground and weeds, it is necessary to first remove the non-target point clouds. Thus, the random sample consensus (RANSAC) algorithm was used to extract the plane model, based on which the ground point cloud was segmented.

Step 4: To reduce external interference and achieve fine measurement, statistical filtering was used to remove outliers. After the noise reduction of point cloud data, the outline of maize plants hardly changed, and it did not affect the extraction of subsequent character parameters.

Step 5: To calculate the phenotypic parameters of individual maize, it is necessary to separate each individual maize from the point cloud. This process was manually completed with the cutting tool of Cloud Compare software.

Step 6: Because maize does not grow perpendicularly to the ground in the growth process, it is necessary to correct the stem axis of the maize first. Rotate the maize point cloud whose stalk axis is not perpendicular to the ground by ±90° around the X axis and Y axis in turn and record the fitting curve formed by the point cloud projection on the X0Z plane. It can be found that when the maize stalk is perpendicular to the ground, the fitting curve formed by its X0Z plane projection tends to be normally distributed. The method combines iterative axis rotation with normal distribution analysis: by systematically rotating the point cloud around the X and Y axes and monitoring the projection’s distribution shape on the X0Z plane, the optimal vertical alignment is achieved when the projection approximates a symmetrical normal distribution, indicating minimized stem tilt. This statistical symmetry criterion ensures objective correction without manual intervention. The axial direction of the maize stem can be effectively corrected using the methods of spatial projection and normal distribution proofreading.

#### 2.3.2. Maize Point Cloud Segmentation

**Figure 5:**
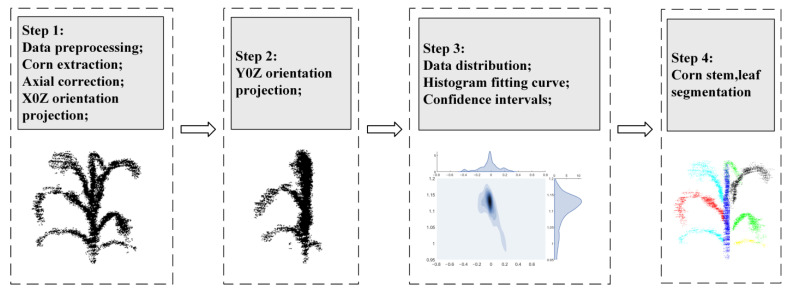
Maize point cloud segmentation.

Maize point cloud segmentation includes two steps: stem extraction and leaf segmentation. Taking a single maize plant as an example, a point cloud segmentation process is shown in Figure 5. The pseudo code of this method is presented in Algorithm 1 and the detailed procedures are as follows.


**Algorithm 1. The Point Cloud Spatial Projection Stem Leaf Segmentation Method**

**Input**
Enter single maize point cloud;
**Output**
Output for maize stem and each leaf on the different colors;
**Step1**
The maize point cloud is projected onto the X0Z and Y0Z planes to obtain the point cloud distribution histograms of the two planes;
**Step2**
Set the confidence interval to obtain the estimation interval of the stem;
**Step3**
Compare the point cloud in the stem area with the original point cloud to obtain the leaf point cloud;
**Step4**
Set cluster tolerance, min cluster size, and max cluster size to obtain point clouds of different leaves.

(1) Stem segmentation comprises four steps: point cloud bidirectional projection, point cloud density curve analysis, confidence interval setting, and stem three-dimensional point cloud reconstruction. The bidirectional projection simplifies 3D stem point cloud analysis by projecting data from two perpendicular directions onto 2D density maps, aiding researchers in visualizing and isolating trunk structures from complex 3D scenes.

Step 1: The point clouds are projected on X0Z and Y0Z planes to obtain two-dimensional images of the front and lateral information of maize.

Step 2: Take the X-axis on the X0Z plane and the Y-axis on the Y0Z plane to generate the point cloud distribution histogram [35], based on which the density curves are fitted.

Step 3: After the maize point cloud is projected on the X0Z and Y0Z planes, the stem point cloud is near the peak value of the density curve. Since the density curve tends to be distributed normally, the estimation interval of the stem can be obtained by calculating confidence intervals on the point cloud density curve.

Step 4: According to the confidence interval, obtain the maximum and minimum values of the X-axis and Y-axis of the maize stalk, compare the original 3D point cloud, and complete the Z-axis data to obtain the 3D point cloud data of the maize stalk.

(2) The point cloud of maize leaves and the individual leaves are segmented by using Euclidean distance. The leaf points are clustered based on Euclidean distance to isolate individual leaves. The Euclidean clustering segmentation algorithm classifies the points whose distance is within a certain threshold into one category. Examine m data points and define a certain affinity between points to divide clustering. The affinity property defined by Euclidean clustering segmentation is Euclidean distance, which is calculated as follows:

(1) $$d(p_{i},q_{i})=\sqrt{\sum_{k=1}^{n}{\left(p_{ik}-q_{ik}\right)}^{2}}$$

where $p_{i},q_{i}\in P$, P indicate the values of the point cluster.

### 2.4. Calculation of PH, LW, and LA of Maize

Based on three-dimensional point cloud data, automatic measurement of PH, LW, and LA of maize plants was realized by using point cloud spatial projection, edge detection, straight-line fitting, and curve fitting. Specific measurement methods of maize morphological parameters are as follows.

#### 2.4.1. Calculation of the PH

**Figure 6:**
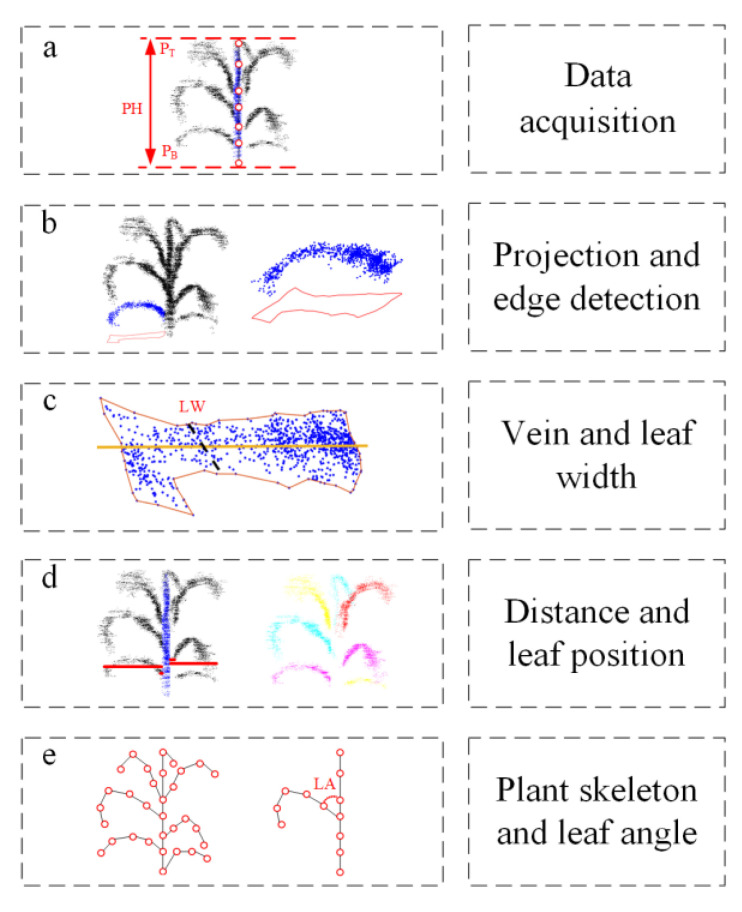
Measurement of morphological parameters of maize. (**a**) Obtain the height of a single maize plant; (**b**) projection and edge detection of single leaf point cloud on X0Y; (**c**) obtain the vein and leaf width of a single leaf; (**d**) measure the distance between different leaves and stems to obtain the leaf position; (**e**) maize plant skeleton and leaf angle.

The key to measuring the PH of maize is to obtain the highest point of the maize plant and determine the connection point between the root and the ground. This process is shown in Figure 6a. Through the segmentation test of maize stems and leaves, we can obtain the point cloud of maize stem, project the point cloud to the X0Z plane, and linearly fit the points on the plane to obtain a straight line on the stem, with the top marked with P_T_ and the bottom marked with P_B_. The height of the maize stalk can be obtained by measuring the length of the straight line, which is expressed by the equation: PH = P_T_ − P_B_.

#### 2.4.2. Calculation of the LW

LW is defined as the length of the widest part of maize leaves, whose calculation is as follows.

Step 1: The leaf point cloud is projected on the X0Y plane.

Step 2: Determining the edges of the data projected on the X0Y plane to obtain the contour of the leaf, as shown in Figure 6b.

Step 3: Fitting a straight line across the points with the least square method to obtain the vein of leaf.

Step 4: Making vertical lines in turn along the vein direction and setting the maximum value of the intersection point between the leaf edge and the vertical line as the LW value, as shown in Figure 6c.

#### 2.4.3. Calculation of the LA

To measure the leaf angle of maize, first obtain the skeleton of maize, and then determine the measurement of the leaf angle of maize through the points on the leaf vein, the intersection of stem and leaf, and the apex of stem.

Step 1: The point clouds of different organs of maize plants can be obtained through the stem and leaf segmentation step. The point clouds of stems and leaves are projected to the X0Z plane, and the points obtained after the projection of stems and leaves are linearly fitted to obtain the skeleton map of maize.

Step 2: In the program measurement, first determine the position of the leaf. The measurement position of the leaf can be divided into two cases: the leaf is located on the left side of the maize stem and the leaf is located on the right side of the maize stalk. Extract the coordinates of the starting point and the last point of each leaf in the X-axis direction and draw a vertical line between the X-axis line of the maize stem and the starting point and the last point of the leaf to obtain the distance between the two points and the stem. If the distance between the starting point of the leaf and the stem is greater than the distance between the final point and the stem, the current leaf is on the left side of the stem. If the distance between the starting point of the leaf and the stem is less than the distance between the final point and the stem, the current leaf is on the right side of the stem, as shown in Figure 6d.

Step 3: Determine the LA values of different leaves and stems by determining the vertex of the maize stem, the intersection point between the stem and the leaf, and the coordinates of the leaf vein points, as shown in Figure 6e.

### 2.5. Evaluation Methodology for LW

In the actual measurement environment, the blade width is measured by taking the midpoint of the leaf vein as the vertical line, and the distance between the two intersection points formed by the vertical line and the blade edge line determines the blade width. Therefore, measurement errors will be introduced in the process of blade multi-frame registration and blade edge extraction, which will affect the accuracy of blade width parameter calculation. Therefore, a method to eliminate this error to a certain extent is introduced, as follows:

(2) $$W_{1}=\eta \times W$$

W is measure width in this paper, $W_{1}$ is corrected width, and η is correction factor.

### 2.6. Evaluating Metrics

To evaluate the accuracy of the proposed method, the true PH, LW, and LA values of maize were measured manually with the collected point cloud. Specifically, the distance between the top and the root of the maize plant is the PH value, the width of the widest part in the middle of maize leaves is selected as the LW value of maize, and the LA value is calculated as the inclination between leaf midvein and stem of maize. We compared the results of manual measurements with those of phenotypic parameters extracted from 3D point clouds. The measurement accuracy was evaluated by mean absolute percentage error (MAPE), root mean square error (RMSE), and correlation coefficient (R^2^) to compare the results. RMSE measures the average prediction error magnitude using the square root of mean squared errors, emphasizing larger deviations. R² quantifies the proportion of variance explained by the model, with values near 1 indicating strong fit. MAPE expresses average error as a percentage of actual values, enabling scale-free interpretation, but failing when actual values approach zero. These were calculated as follows:

(3) $$R^{2}=1-\frac{\sum_{l=1}^{m}(v_{l}-{v^{\prime }}_{l}{)}^{2}}{\sum_{l=1}^{m}{\left(v_{l}-{\bar{v}}_{l}\right)}^{2}}$$

(4) $$\mathrm{RMSE}=\sqrt{\frac{1}{m}\sum_{l=1}^{m}{\left(v_{l}-{v^{\prime }}_{l}\right)}^{2}}$$

(5) $$M\mathrm{APE}=\frac{1}{m}\sum_{t=1}^{m}\frac{\left|v_{l}-v^{\prime }\right|}{v_{l}}\times 100\%$$

m: Indicates the number of objects to compare.

$v_{l}$: Indicates the value of manual measurement results.

$v^{\prime }$: Represents the value of phenotypic parameters extracted from 3D point cloud segmentation results.

${\bar{v}}_{l}$: Indicates the average of manual measurements.

## 3. Results and Analysis

### 3.1. Results of Stem–Leaf Segmentation

**Figure 7:**
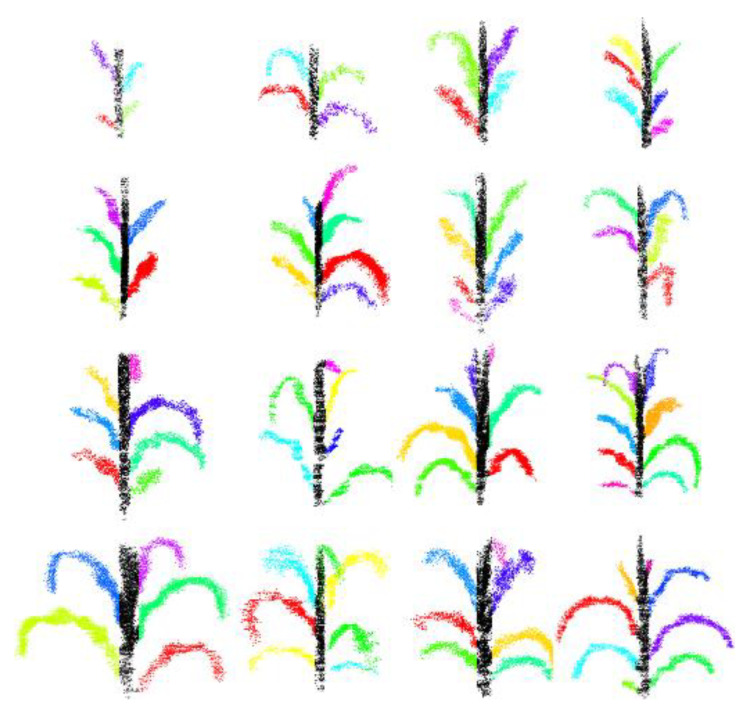
Maize stem and leaf segmentation at different stages. Dark black: maize stem, other colors: maize leaves.

The organ segmentation results of the maize point cloud were verified by visual segmentation and quantitative indicators. Figure 7 presents 16 distinct samples collected from different maize growth stages, arranged in ascending order of leaf number and size. Each leaf is color-coded for segmentation, and the image demonstrates precise delineation of both leaves and stems, effectively illustrating their three-dimensional posture and morphological architecture.

### 3.2. Evaluation Performance of the PH Value

**Figure 8:**
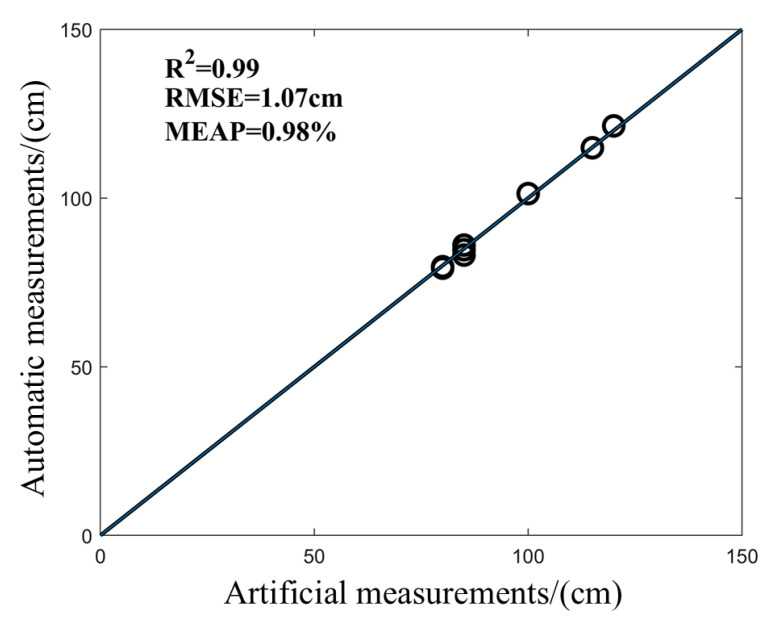
Determination of PH value of maize.

The PH value of Jinyunuo 856 was automatically extracted by the program and compared with the result of artificial point cloud measurement. As shown in Figure 8, the PH of maize is automatically measured. Based on the results, the R^2^ value is greater than 0.99, indicating an excellent match between values obtained with the proposed method and those with the manual measurements. In addition, the RMSE is 1.07 cm and MAPE is less than 0.98%. The results show that this method has high precision in measuring the PH value of maize, indicating its possible application in the real-world breeding process for maize plants.

### 3.3. Evaluation of the LW Value

**Figure 9:**
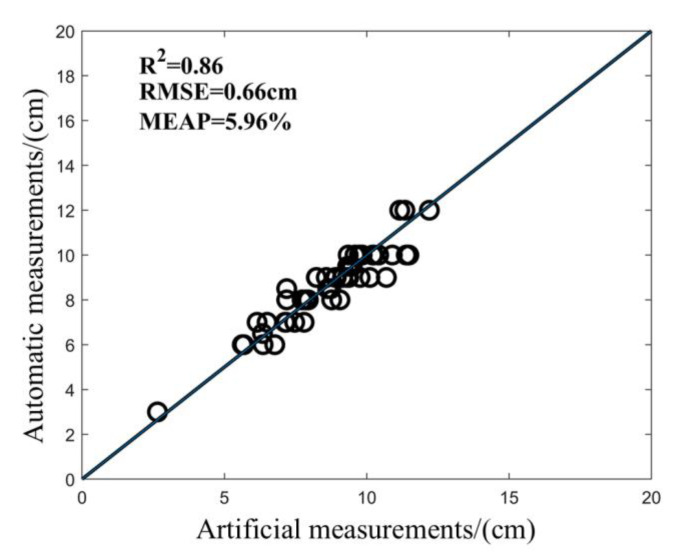
Determination of LW value of maize.

The LW of Jinyunuo 856 was measured automatically and correctly by this program and compared with the artificial point cloud measurement results. As shown in the figure, the LW of maize was measured automatically (Figure 9). R2 is greater than 0.86; RMSE is controlled within 0.66 cm; MAPE is not more than 5.96%; and LW automatic measurement accuracy is not less than 86%. The results show that this method has high accuracy in measuring the LW of maize, and the measured value of the algorithm is consistent with the real value measured by artificial point cloud.

### 3.4. Evaluation of the LA Value

**Figure 10:**
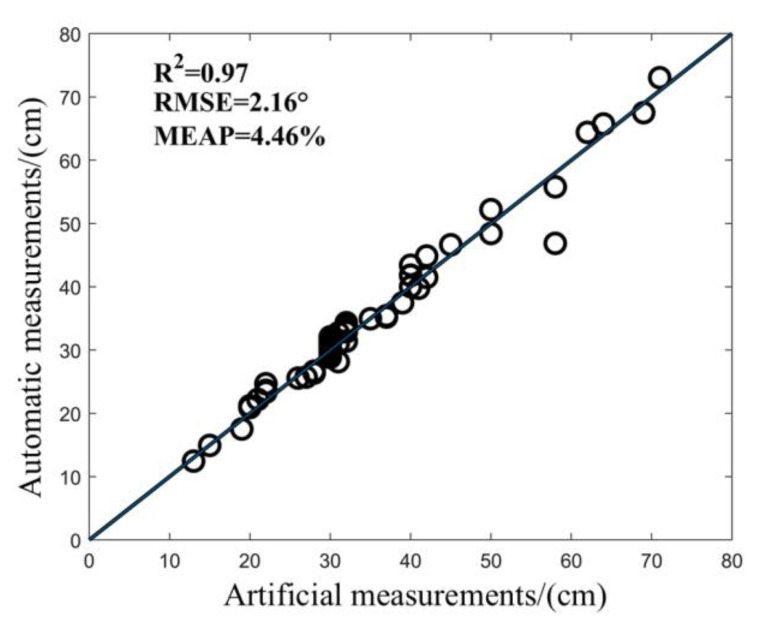
Determination of LA value of maize.

The program was used to automatically extract the LA of Jinyunuo 856 and compared with the artificial point cloud measurement results. As shown in Figure 10, the LA value of maize was automatically determined. R2 is greater than 0.97; RMSE is controlled within 2.16°; MAPE is not more than 4.46%; and the accuracy of automatic measurement of LA is not less than 97%. The results show that this method has high accuracy in measuring the LA of maize, and the measured value of the algorithm is consistent with the real value measured by artificial point cloud. It is feasible to use VLP-16 lidar to collect a maize point cloud and measure the LA of maize. This method can replace manual measurement, improve measurement accuracy and efficiency, and reduce the influence of human factors.

## 4. Discussion

In this study, the VLP-16 lidar sensor was used to obtain the point cloud data of maize plant in the middle and late growth stages. The measured values of PH, LW, and LA were obtained by point cloud preprocessing and stem–leaf segmentation. In addition, the automatically measured values were highly consistent with the manual measurements, indicating the feasibility of using VLP-16 lidar to measure phenotypic parameters of maize plants. Since critical parameters of maize can be efficiently measured with the proposed method, the plant growth process can be quantitatively analyzed.

Compared with the precision of TLS maize phenotype determination, Wei et al. realized the measurement of PH through the maximum height of plant points from the ground. The RMSE of PH was 1.3 cm [23]. In this study, the PH value RMSE of Jinyunuo 856 was 1.07 cm and the RMSE was increased by 0.23 cm. Jin et al. achieved LW measurement by selecting the widest part of the leaf point cloud projected on the Y0Z plane. The RMSE of PH was 1 cm [27]. In this study, the LW value RMSE of Jinyunuo 856 was 0.66 cm, and RMSE was decreased by 0.34 cm. Liu et al. measured the coefficient R^2^ of leaf angle as 0.82 [29]. In this study, the determination coefficient R^2^ of LA value of Jinyunuo 856 is 0.97, which is increased by 0.15.

Compared with the efficiency of TLS in maize phenotype measurement, Miao et al. collected data for 10 min, which resulted in a change in maize leaf shape and measurement error when collecting point cloud data with long intervals [28]. In addition, when collecting data in the field, some environmental factors will lead to changes in the morphology of maize plants, resulting in a big difference between the collected three-dimensional point cloud data of maize plants and the actual three-dimensional morphology. VLP-16 lidar is loaded in the plant protection vehicle, which can record the phenotypic information of maize in multiple directions and angles without the need to collect maize point cloud data at a fixed position. The single acquisition time is shorter and the efficiency is higher.

Furthermore, the middle and late growth stages of maize are vegetative growth stages, and the three-dimensional morphology of plants is similar, so the general measurement method can be adopted. During the reproductive growth period, the morphology of maize plants changed significantly in 3D: the male ears of maize were completely pulled out, the female ears became larger, and the lower leaves began to wither and attach to the plants. Further research is needed to determine whether the measurement method is appropriate. The simple and efficient projection method is used to quickly extract the stem point cloud in the segmentation of maize stems and leaves. However, due to the artificial setting of the stem confidence interval and confidence interval overlap, some leaf points may be misclassified as stem points, introducing errors, resulting in errors between the measured LW and LA values and the true ones. Further research is needed to improve the accuracy of stem extraction. In addition, other phenotypic parameters of maize, such as stem width, leaf length, leaf area, and so on, can also be measured using three-dimensional point cloud data. In future research, this technique will be extended to the measurement of other morphological parameters.

## 5. Conclusions

This study presents an automated measurement method using VLP-16 lidar sensors for determining maize phenotypic parameters. Focusing on maize plants during middle–late growth stages, the self-developed platform achieves rapid determination of PH, LW, and LA through three key innovations: (1) stem–leaf segmentation combining spatial projection with normal distribution; (2) PH calculation via vertical distance between plant apex and ground contact point (RMSE = 1.07 cm, MAPE = 0.99%); and (3) LW and LA measurement using Euclidean distance-based leaf identification with linear fitting and spatial distance algorithms (LW: RMSE = 0.66 cm, MAPE = 5.96%; LA: RMSE = 2.16°, MAPE = 4.46%). The proposed non-destructive method demonstrates potential to replace manual destructive sampling for maize breeding applications. It should be noted that while validated for middle–late growth stages, the method’s applicability to reproductive phases requires further investigation.

## Figures and Tables

**Table 1:** Performance parameters of VLP-16 lidar.

Specifications	Parameters
Laser line number	16
Measurement range	0.5–100 m
Measurement accuracy	±3 cm
Horizontal field of view	360°
Vertical field of view	30° (−15°–+15°)

## Data Availability

Data are contained within the article.
